# Association Between Frailty and Management and Outcomes of Acute Myocardial Infarction Complicated by Cardiogenic Shock

**DOI:** 10.1016/j.jacadv.2024.100949

**Published:** 2024-05-17

**Authors:** Yasser Jamil, Dae Yong Park, Sunil V. Rao, Yousif Ahmad, Nikhil V. Sikand, Hayden B. Bosworth, Theresa Coles, Abdulla A. Damluji, Michael G. Nanna, Marc D. Samsky

**Affiliations:** a Department of Medicine, Yale School of Medicine, New Haven, Connecticut, USA; b Department of Medicine, Cook County Health, Chicago, Illinois, USA; c Grossman School of Medicine, New York University Langone Health System, New York University, New York, New York, USA; d Section of Cardiovascular Medicine, Yale School of Medicine, New Haven, Connecticut, USA; e Division of General Internal Medicine, Department of Medicine, Duke University, Durham, North Carolina, USA; f Department of Psychiatry and Behavioral Health Sciences, Duke University School of Nursing, Durham, North Carolina, USA; g Department of Population Health Sciences, Duke University School of Medicine, Durham, North Carolina, USA; h Johns Hopkins University School of Medicine, Baltimore, Maryland, USA; i Inova Center of Outcomes Research, Falls Church, Virginia, USA

**Keywords:** acute myocardial infarction, cardiogenic shock, frailty

## Abstract

**Background:**

Cardiogenic shock (CS) in the setting of acute myocardial infarction (AMI) is associated with high morbidity and mortality. Frailty is a common comorbidity in patients with cardiovascular disease and is also associated with adverse outcomes. The impact of preexisting frailty at the time of CS diagnosis following AMI has not been studied.

**Objectives:**

The purpose of this study was to examine the prevalence of frailty in patients admitted with AMI complicated by CS (AMI-CS) hospitalizations and its associations with in-hospital outcomes.

**Methods:**

We retrospectively analyzed the National Inpatient Sample from 2016 to 2020 and identified all hospitalizations for AMI-CS. We classified them into frail and nonfrail groups according to the hospital frailty risk score cut-off of 5 and compared in-hospital outcomes.

**Results:**

A total of 283,700 hospitalizations for AMI-CS were identified. Most (70.8%) occurred in the frail. Those with frailty had higher odds of in-hospital mortality (adjusted OR [aOR]: 2.17, 95% CI: 2.07 to 2.26, *P* < 0.001), do-not-resuscitate status, and discharge to a skilled nursing facility compared with those without frailty. They also had higher odds of in-hospital adverse events, including intracranial hemorrhage, gastrointestinal hemorrhage, acute kidney injury, and delirium. Importantly, AMI-CS hospitalizations in the frail had lower odds of coronary revascularization (aOR: 0.55, 95% CI: 0.53-0.58, *P* < 0.001) or mechanical circulatory support (aOR: 0.89, 95% CI: 0.85-0.93, *P* < 0.001). Lastly, hospitalizations for AMI-CS showed an overall increase from 53,210 in 2016 to 57,065 in 2020 (*P* trend <0.001), with this trend driven by a rise in the frail.

**Conclusions:**

A high proportion of hospitalizations for AMI-CS had concomitant frailty. Hospitalizations with AMI-CS and frailty had higher rates of in-hospital morbidity and mortality compared to those without frailty.

## Central Illustration

**Figure:**
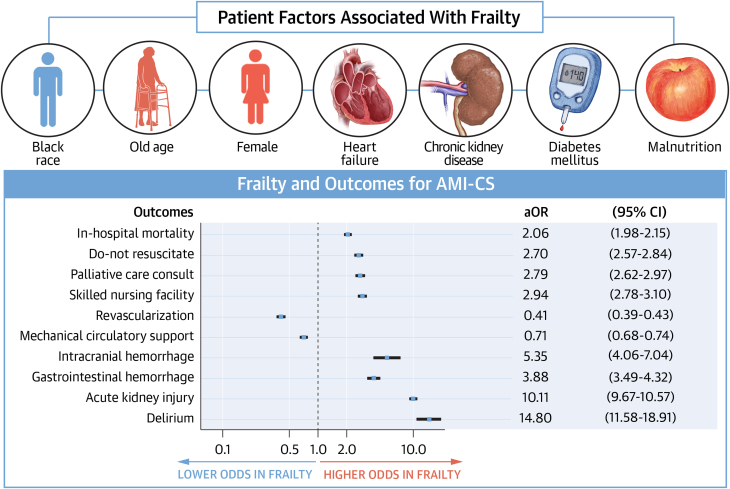

Frailty is an age-related syndrome characterized by a reduced physiologic reserve and increased vulnerability to external and internal stressors.^1^ It is associated with poor health outcomes, increased mortality, morbidity, and a reduced quality of life (QoL).^2^^,^^3^ Increased attention has been given to the bidirectional association between frailty and cardiovascular diseases (CVD) and its correlation with a heightened risk of cardiovascular events.^4^ Frailty increases the risk of adverse cardiovascular outcomes; conversely, CVD increases the risk of being frail. This bidirectional association has been seen in patients with acute myocardial infarction (AMI) and several other cardiovascular conditions.^5^, ^6^, ^7^, ^8^ However, frailty is not well characterized in patients with cardiogenic shock (CS), which is a highly lethal condition that can develop after AMI and is independently associated with poor outcomes.^9^

CS is the leading cause of death in patients with AMI and is associated with multimorbidity, reduced QoL, and physical disability.^9^ In the broader general population of patients with AMI, frail individuals are more likely to undergo conservative management, with lower utilization of revascularization therapies and advanced hemodynamic support.^10^^,^^11^ However, the prevalence of frailty in this vulnerable population and clinical characteristics of frail patients with AMI complicated by CS (AMI-CS) have not been characterized. Furthermore, the relationship between frailty and clinical outcomes among the highest-risk subset of individuals suffering from CS as a complication of AMI remains unknown. Therefore, we sought to define the prevalence of frailty in patients admitted to AMI-CS hospitalizations and examine its associations with in-hospital outcomes.

## Methods

### Data source

We retrospectively analyzed the National Inpatient Sample, the largest all-payer inpatient care database in the United States, developed for the Healthcare Cost and Utilization Project (HCUP) and sponsored by the Agency for Healthcare Research and Quality.^12^ When weights are applied to the 20% of participating hospitals across 49 participating states, each year of HCUP contains data on approximately 35 million hospitalizations, which can be used to identify, track, and analyze health care utilization, access, costs, quality, and outcomes. The database has been devised to account for more than 97% of admissions occurring nationally. The HCUP is devoid of state, hospital, and patient identifiers to guarantee patient confidentiality, and since all hospital encounters are strictly deidentified, our study was exempt from the purview of our institutional review board. HCUP is open to the public and can be accessed through its public website.^12^ This study was exempt from ethics approval as publicly available deidentified data from the National Inpatient Sample were used.

### Study population and covariates

We collected all hospitalizations for AMI-CS in either primary or secondary diagnoses from the HCUP years 2016 to 2020. We then excluded hospitalizations under 18 years old and entries containing missing data on demographics, hospital characteristics, primary payer, median income,^12^ day of hospitalization, in-hospital mortality, and length of hospital stay (LOS). After the application of inclusion and exclusion criteria, we extracted data on demographics (sex, age, race), hospital characteristics (region, bed size, urban location), primary payer, median income, and day of hospitalization (weekday, weekend), all of which are present in the original databases. In each hospitalization, we examined the presence of many comorbidities, as shown in Table 1. Using International Classification of Diseases-10th Revision Clinical Modification (ICD-10-CM) codes (Supplemental Table 1), we calculated hospital frailty risk score (HFRS), a validated measure of clinical frailty, for each hospitalization. In short, HFRS is derived by awarding each prespecified ICD-10-CM code with a different number of points and then aggregating all the points awarded.^13^ We defined the presence of frailty as having an HFRS of at least 5, consistent with the definition used by many previous studies.^14^, ^15^, ^16^ AMI-CS hospitalizations were grouped into those with and without frailty. Clinical presentation was classified into ST-segment elevation myocardial infarction (STEMI) and non-STEMI (NSTEMI). All the comorbidities and procedural data used in our study were established on the basis of ICD-10-CM and International Classification of Diseases-10th Revision, Procedural Coding System codes, respectively. These codes can be found in Supplemental Table 2.

**Table 1:** Baseline Characteristics of Acute Myocardial Infarction Complicated by Cardiogenic Shock Hospitalizations With and Without Frailty

	Frailty (+)	Frailty (−)	*P* Value
Number of hospitalizations	200,970	82,800	
Male	63.0	65.6	<0.001
Age, y	69.5 ± 12.6	66.2 ± 12.6	<0.001
Race^a^			<0.001
White	71.1	74.6	
Black	10.9	7.8	
Hispanic	9.7	8.9	
Asian	4.0	3.8	
AI/AN	0.7	0.7	
Other	3.6	4.3	
Comorbidities^b^			
Smoking	37.0	43.5	<0.001
Hypertension	17.0	33.9	<0.001
Diabetes mellitus	45.4	36.9	<0.001
Hyperlipidemia	47.8	57.8	<0.001
Obesity	16.3	16.5	0.660
Heart failure	70.0	50.9	<0.001
Chronic ischemic heart disease	14.6	10.0	<0.001
Atrial fibrillation	33.9	24.3	<0.001
Valvular heart disease	13.2	11.4	<0.001
Peripheral artery disease	10.3	8.2	<0.001
Previous PCI	1.1	1.3	0.104
Previous CABG	7.5	6.5	<0.001
Previous stroke	10.4	5.8	<0.001
Previous pacemaker	2.3	1.9	0.003
COPD	22.0	16.7	<0.001
Pulmonary hypertension	10.9	6.5	<0.001
Chronic kidney disease	40.0	12.0	<0.001
End-stage renal disease	9.1	3.0	<0.001
Liver cirrhosis	2.9	1.5	<0.001
History of malignancy	7.3	7.1	0.495
Deficiency anemia	5.8	2.6	<0.001
Malnutrition	11.6	3.6	<0.001
Dementia	7.8	1.1	<0.001
Major depression	0.6	0.4	<0.001
HFRS^c^	9.3	2.6	<0.001
Hospital characteristics			
Hospital region			<0.001
Northwest	15.8	17.6	
Midwest	21.6	19.6	
South	40.6	42.1	
West	21.9	20.7	
Hospital bed-size			0.020
Small	14.1	14.4	
Medium	27.9	28.8	
Large	58.0	56.8	
Urban location			<0.001
Rural	4.3	5.5	
Urban nonteaching	18.2	20.1	
Urban teaching	77.5	74.4	
Primary payer			<0.001
Medicare	66.2	53.6	
Medicaid	9.2	9.8	
Private insurance	18.0	27.2	
Self-pay	3.6	5.7	
No charge	0.3	0.3	
Others	2.7	3.3	
Median income			0.615
Quartile 1	30.1	29.6	
Quartile 2	26.9	27.1	
Quartile 3	23.8	24.1	
Quartile 4	19.2	19.3	
Clinical presentation			<0.001
STEMI	43.2	62.2	
NSTEMI	56.8	37.8	
Day of hospitalization			0.963
Weekday	73.2	73.2	
Weekend	26.8	26.8	

Values are % or mean ± SD unless otherwise indicated.

AI/AN = American Indian/Alaska Native; CABG = coronary artery bypass graft; COPD = chronic obstructive pulmonary disease; CS = cardiogenic shock; HFRS = Hospital Frailty Risk Score; NSTEMI = non-ST-segment elevation myocardial infarction; PCI = percutaneous coronary intervention; STEMI = ST-segment elevation myocardial infarction.

a Race in the studied database is provided by the Healthcare Cost and Utilization Project partner organizations, and the protocol whereby race was determined is not specifically mentioned but rather left to the discretion of the data source.

b The variables used to define the comorbidities are shown in Supplemental Table 2.

c The variables used to derive HFRS are shown in Supplemental Table 1.

### Study Outcomes

The primary outcome of interest was in-hospital mortality. Secondary outcomes included do-not-resuscitate status (DNR), palliative care consult, discharge to a skilled nursing facility, coronary revascularization, use of mechanical circulatory support (MCS), intracranial hemorrhage, gastrointestinal hemorrhage, acute kidney injury (AKI), delirium, LOS, and total hospital cost. Coronary revascularization was defined as having undergone percutaneous coronary intervention or coronary artery bypass graft. Percutaneous coronary intervention was defined as the implantation of a drug-eluting stent, bare-metal stent, or balloon angioplasty. Mechanical circulatory support was defined as using an intra-aortic balloon pump, percutaneous left ventricular assist device, durable left ventricular assist device, or extracorporeal membranous oxygenation. Total hospital cost was calculated by multiplying the total hospital charge with the cost-to-charge ratios from separate files obtained via HCUP.^17^

### Statistical analysis

In all statistical analyses, we applied weights of hospital-level discharge to produce results representative of national estimates. We compared categorical and continuous covariates in the baseline characteristics using the chi-square test and the Student’s *t* test, respectively. We examined the trend of the number of hospitalizations from 2016 to 2020 using the Jonckheere-Terpstra test. When selecting covariates to adjust statistical models, we first examined all baseline characteristics in a correlation matrix to ensure that no 2 covariates were highly correlated, as defined by a Pearson correlation coefficient >0.80. Secondly, we investigated for multicollinearity using variance inflation factor and tolerance, whose cut-offs were 3 and 0.1, respectively. Thirdly, we ran collinearity diagnostics for an eigensystem analysis of covariance to double-check the absence of multicollinearity. After addressing multicollinearity, we used stepwise selection on a multivariable logistic regression model to select covariates to adjust when comparing AMI-CS with frailty vs without frailty. The model with covariates that produced the least Akaike information criterion was selected. The following 21 covariates were selected: age, sex, race, smoking, diabetes mellitus, hyperlipidemia, obesity, heart failure, chronic ischemic heart disease, atrial fibrillation, peripheral artery disease, previous coronary artery bypass graft, previous stroke, pulmonary hypertension, end-stage renal disease, liver cirrhosis, deficiency anemia, malnutrition, dementia, major depression, and STEMI.

To compare primary and secondary outcomes, which were binary, we used both simple and multivariable logistic regression to produce crude odds ratios and adjusted ORs (aORs) with respective 95% CI. We utilized linear regression when comparing continuous secondary outcomes. We conducted a sensitivity analysis in which we included AMI-CS managed with either revascularization or MCS. We also performed 2 subgroup analyses: one stratified to younger (age <65 years) and older (age ≥65 years) adults, and one stratified to STEMI and NSTEMI. Moreover, after stratifying into frail and nonfrail groups, we looked at the impact of revascularization, MCS, or either one of them on the in-hospital outcomes of AMI-CS using the same analytical methodology. Finally, to examine the correlation of frailty score with in-hospital outcomes, the same multivariable logistic regression models were used to calculate the log-odds of each of the in-hospital outcomes, which were then graphed against the frailty score using cubic splines with smoothing parameter set at 0.7. Afterward, simple linear regression was used to assess for linear trends. All tests were 2-sided, and *P* values <0.05 were considered significant. Data curation and all statistical analyses were conducted using SAS, version 9.4 (SAS Institute). Production of figures were assisted by R version 4.2.3 (R Foundation for Statistical Computing, Vienna, Austria).

## Results

**Figure 1:**
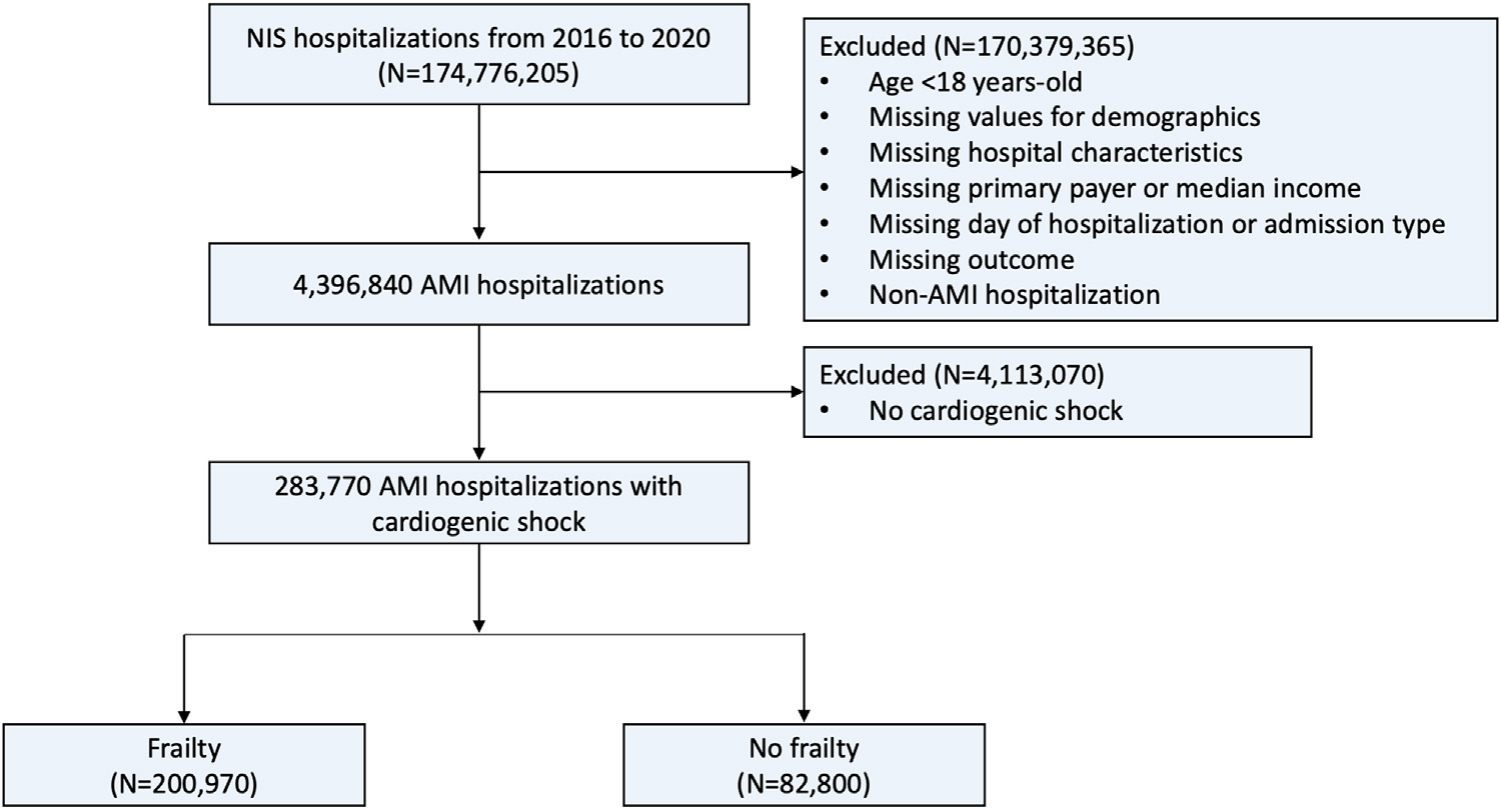
**Flowchart of This Study** The flowchart illustrates the patient selection process used in this study. AMI = acute myocardial infarction; NIS = National Inpatient Sample.

**Figure 2:**
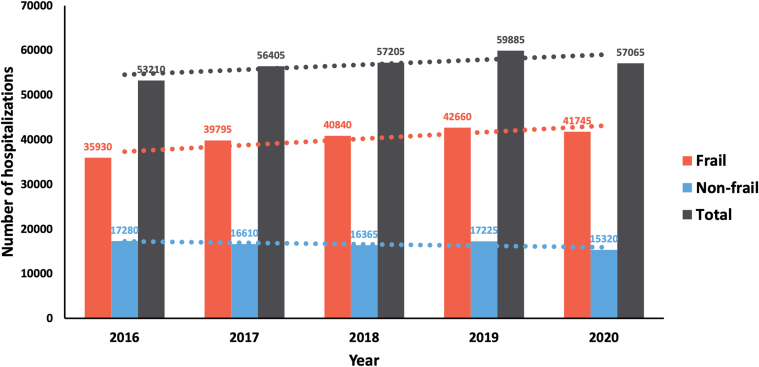
**Trend of Acute Myocardial Infarction Hospitalizations With Cardiogenic Shock** The bar graphs show the number of hospitalizations for AMI with cardiogenic shock from the year 2016 to 2020 in the frail (red), nonfrail (blue), and both (gray). The lines of best fit for each of the groups are shown in dotted lines. AMI = acute myocardial infarction.

A total of 4,396,840 AMI hospitalizations were identified, of which 283,770 (6.5%) also had CS (Figure 1). Among the AMI-CS hospitalizations, 200,970 (70.8%) occurred in the frail, while 82,800 (29.2%) occurred in those without frailty. The frail group was slightly older than the nonfrail group (age 69.5 years vs 66.2 years, *P* < 0.001) and had a higher proportion of individuals identified as of black race and Hispanic ethnicity (Table 1). In the frail group, the proportions of diabetes mellitus, heart failure, atrial fibrillation, previous stroke, chronic obstructive pulmonary disease, chronic kidney disease, deficiency anemia, malnutrition, and dementia were significantly higher. However, the proportions of smoking, hypertension, and hyperlipidemia were lower (Table 1). The majority of the frail group presented with NSTEMI (56.8%), in contrast to STEMI being the major clinical presentation for the nonfrail group (62.2%). The overall number of hospitalizations for AMI-CS increased from 53,210 in 2016 to 57,065 in 2020 (*P* trend <0.001) (Figure 2). This increasing trend was mediated by an increasing number of AMI-CS hospitalizations in the frail (*P* trend <0.001), in contrast to the decreasing number of AMI-CS hospitalizations in the nonfrail (*P* trend <0.001).

AMI-CS hospitalizations with frailty had significantly higher unadjusted rates of in-hospital mortality (40.0% vs 24.5%, *P* < 0.001) compared with those without frailty (Table 2). The odds of in-hospital mortality were significantly higher even after adjusting for confounders (aOR: 2.17, 95% CI: 2.07-2.26, *P* < 0.001). The frail group had higher odds of having DNR status or receiving a palliative care consult (Central Illustration). They also had higher odds of disposition to a skilled nursing facility, intracranial hemorrhage, gastrointestinal hemorrhage, AKI, and delirium. However, they had lower odds of undergoing coronary revascularization or receiving MCS. The LOS in the frail group was significantly higher (adjusted mean difference 3.91 days, 95% CI: 3.71-4.10, *P* < 0.001) and more expensive (adjusted mean difference $17,705, 95% CI: 16,629-18,781, *P* < 0.001).

**Table 2:** Comparison of Outcomes in Acute Myocardial Infarction Complicated by Cardiogenic Shock With and Without Frailty

Outcome	Frailty (+)	Frailty (−)	Crude OR (95% CI)	*P* Value	Adjusted OR (95% CI)^a^	*P* Value
In-hospital mortality	40.0	24.5	2.06 (1.98-2.15)	<0.001	2.17 (2.07-2.26)	<0.001
Do not resuscitate	29.7	13.6	2.70 (2.57-2.84)	<0.001	2.36 (2.24-2.49)	<0.001
Palliative care consult	21.1	8.8	2.79 (2.62-2.96)	<0.001	2.40 (2.25-2.56)	<0.001
Skilled nursing facility	26.1	10.7	2.94 (2.78-3.10)	<0.001	2.23 (2.10-2.35)	<0.001
Revascularization	47.4	68.8	0.41 (0.39-0.43)	<0.001	0.55 (0.53-0.58)	<0.001
MCS	34.0	42.1	0.71 (0.68-0.74)	<0.001	0.89 (0.85-0.93)	<0.001
Intracranial hemorrhage	1.7	0.3	5.35 (4.06-7.04)	<0.001	5.73 (4.31-7.61)	<0.001
Gastrointestinal hemorrhage	8.5	2.4	3.88 (3.49-4.32)	<0.001	3.64 (3.26-4.08)	<0.001
Acute kidney injury	71.4	19.8	10.11 (9.67-10.57)	<0.001	11.06 (10.55-11.59)	<0.001
Delirium	5.6	0.4	14.80 (11.58-18.90)	<0.001	13.20 (10.32-16.87)	<0.001
Length of stay, days	11.0 ± 12.3	3.5 ± 3.6	5.27 (5.07-5.47)^b^	<0.001	3.91 (3.71-4.10)^c^	<0.001
Total hospital cost, $	58,075 ± 66,153	38,290 ± 37,921	19,785 (18,712-20,859)^b^	<0.001	17,705 (16,629-18,781)^c^	<0.001

Values are % or mean ± SD unless otherwise indicated.

CABG = coronary artery bypass graft; MCS = mechanical circulatory support.

a Adjusted for age, sex, race, smoking, diabetes mellitus, hyperlipidemia, obesity, heart failure, chronic ischemic heart disease, atrial fibrillation, peripheral artery disease, previous CABG, previous stroke, pulmonary hypertension, end-stage renal disease, liver cirrhosis, deficiency anemia, malnutrition, dementia, major depression, and ST-segment elevation myocardial infarction.

b Crude mean difference with a 95% CI.

c Adjusted mean difference with a 95% CI.

**Central Illustration:**
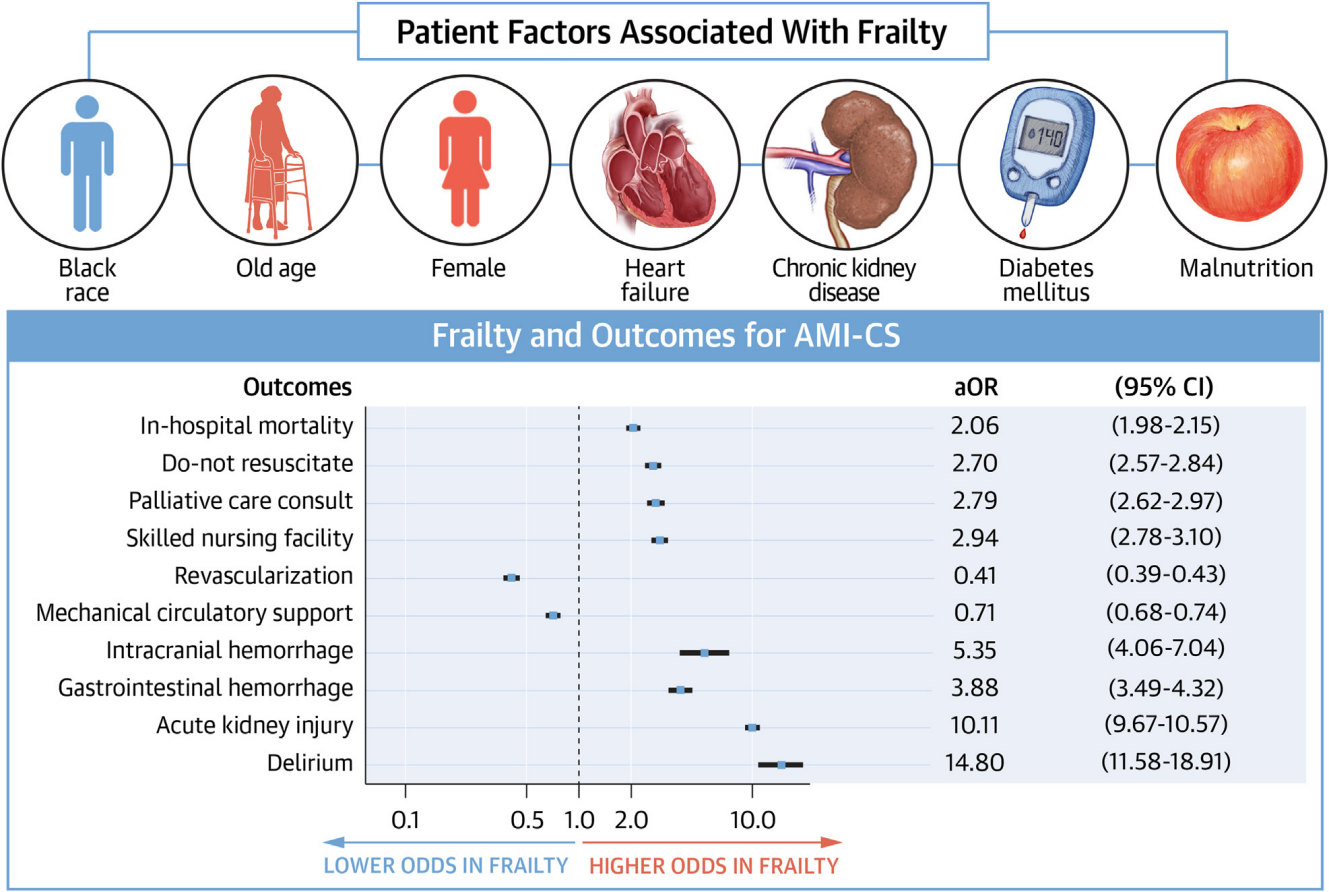
**Baseline Demographics and Hospital Outcomes of Frail Adults With AcuteMyocardial Infarction Complicated With Cardiogenic Shock**

**Figure 3:**
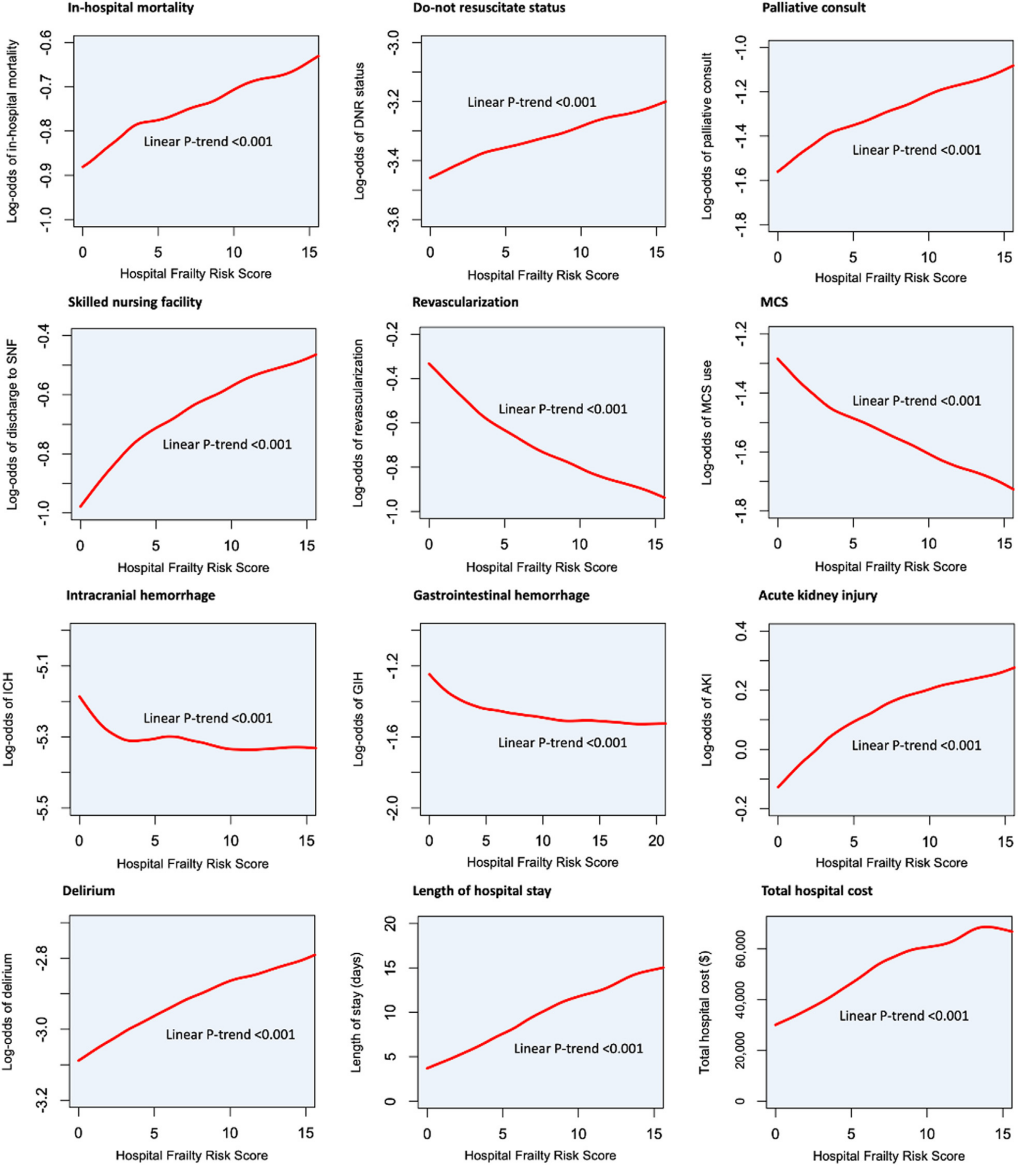
**Spline Curves Showing Association of HFRS With in-Hospital Outcomes** The spline curves illustrate the correlation between HFRS and log-odds of various adverse in-hospital outcomes. AKI = acute kidney injury; DNR = do not resuscitate; GIH = gastrointestinal hemorrhage; ICH = intracranial hemorrhage; MCS = mechanical circulatory support; SNF = skilled nursing facility.

Sensitivity analysis of AMI-CS managed with either revascularization or mechanical circulatory support showed similar results of increased in-hospital mortality, ICH, gastrointestinal bleed, and longer length of stay (Supplemental Table 3). Subgroup analyses stratified by younger (n = 104,025) and older adults (n = 179,745) revealed similar results in both AMI-CS hospitalizations occurring in ages <65 years and ≥65 years (Supplemental Table 4). The results were largely similar in another subgroup analysis stratified to STEMI and NSTEMI, except for the similar rates of MCS use regardless of frailty in STEMI hospitalizations (Supplemental Table 5). Revascularization was associated with lower odds of in-hospital mortality in frail and nonfrail groups. However, MCS was associated with lower odds of in-hospital mortality only in the frail group (aOR: 0.91, 95% CI: 0.87-0.96, *P* < 0.001). (Supplemental Table 6). Spline curves showed a positive correlation between HFRS and log-odds of in-hospital mortality, DNR status, palliative consult, discharge to a skilled nursing facility, AKI, and delirium (Figure 3). On the other hand, a negative correlation was seen between HFRS, log odds of revascularization, and MCS. All the linear trends were significant.

## Discussion

This nationally representative study assessed the association between frailty, clinical management, and clinical outcomes in AMI-CS hospitalizations. First, the prevalence of frailty in AMI-CS hospitalization is high, with nearly three-quarters being frail. Second, frailty during AMI-CS hospitalization was associated with higher rates of mortality and in-hospital complications such as bleeding, delirium, and longer LOS than those with nonfrail status. Third, frailty in AMI-CS was associated with a lower likelihood of coronary artery revascularization and temporary MCS devices. Lastly, among the frail group, those who underwent coronary revascularization experienced lower in-hospital mortality. However, mortality benefit from MCS was observed specifically in the frail group and not the nonfrail.

Our findings suggest that frailty is more prevalent in adults hospitalized with AMI-CS than in other cardiovascular conditions. Up to 70% of AMI-CS hospitalizations had frailty, which could be attributed to the combined burden of 2 acute CVD diagnoses (AMI and CS). Furthermore, on the opposite end, the reduced overall reserve in frail individuals makes them more susceptible to CS following AMI.^18^, ^19^, ^20^, ^21^ Previous studies reported varying rates of frailty in CVD. For example, frailty prevalence in patients with acute CVD has been reported to range from 24 to 86%, varying according to the underlying CVD process.^6^^,^^22^, ^23^, ^24^, ^25^ The variability in the reported rates can be attributed to multiple factors, such as the lack of standardized diagnosis, assessment tools for frailty, and the differences in underlying CVD severity.^4^ Furthermore, our study revealed that hospitalizations for AMI-CS with concomitant frailty were associated with a 40% in-hospital mortality rate. Similar to the current study, previous registries, such as the LONGEVO-SCA (Impact of Frailty and Other Geriatric Syndromes on the Management and Vital Prognosis of the Elderly with Acute Coronary Syndrome without ST Segment Elevation) registry and TRILOGY ACS (Targeted Platelet Inhibition to Clarify the Optimal Strategy to Medically Manage Acute Coronary Syndromes) trial, demonstrated up to 40% in-hospital mortality rates in patients with ACS who were considered frail.^23^^,^^26^^,^^27^ The high mortality rates among frail adults may be attributed to a higher number of comorbidities and higher rates of sarcopenia, reduced physiological reserve, and diminished rehabilitation potential.^28^ However, it remains unknown if interventions that directly target frailty can improve outcomes in AMI-CS complicated by frailty.

This study showed that frailty was associated with negative outcomes across age groups (>65 vs <65 years), emphasizing that the adverse effects of frailty in those with AMI-CS extend across the aging spectrum. This finding across age groups is noteworthy because some may assume that frailty is exclusively observed in older adults, whereas younger adults are also susceptible to frailty. Most studies reporting frailty, however, include adults above 65 years of age and sometimes even higher, limiting the generalizability of the evidence in the frail younger population.^29^

In terms of treatment strategies, this study revealed that frailty during AMI-CS hospitalization was associated with a lower likelihood of receiving coronary artery revascularization (47.4% vs 68.6%, *P* < 0.001) or MCS (34.0% vs 42.1%, *P* < 0.001). Consistent with prior research, the CONCORDANCE and ACTION registries demonstrated that older adults with frailty and AMI-CS had a lower likelihood of receiving invasive cardiac care (ie, percutaneous coronary intervention), as low as 6%.^20^^,^^30^, ^31^, ^32^ Interestingly, in the current study, among the frail group, those who underwent coronary artery revascularization had lower hospital mortality compared to those who did not (aOR: 0.44, 95% CI: 0.42-0.46, *P* < 0.001). Other studies similarly demonstrated benefit, a 21% reduction in mortality rates among those who received revascularization.^33^, ^34^, ^35^, ^36^ Regarding MCS, it is unsurprising that frail adults are managed more conservatively. Although it is uncertain whether MCS can decrease long-term mortality rates as well as reverse frailty, this study noted a hospital mortality benefit in the frail group who had MCS (aOR: 0.91, 95% CI: 0.87-0.96, *P* < 0.001), which was not seen in the nonfrail group. Most landmark trials have excluded frail adults, such as in the EURO-SHOCK, or failed to measure and/or report frailty with their interventions.^37^ Therefore, the precise impact of given therapies on frail populations remains unclear.

### Study Limitations

There are several limitations to this study. This is a retrospective analysis, which is prone to bias and confounders. The data used in the study is based on an administrative dataset, which has inherent limitations. The HFRS is based on ICD codes, which may result in inaccurate coding and inaccuracies in the diagnosis of frailty.^13^^,^^38^ Patients with CS are historically not reported in cardiovascular quality improvement registries, and markers of frailty are also not reported. Therefore, national datasets such as the HCUP provide a unique opportunity to complete these analyses. Although the frailty score utilized in this study, known as the HFRS, has been validated and demonstrated a reasonable degree of overlap with the Fried and Rockwood Frailty Index and shows consistent correlations with frailty indices in other studies.^13^^,^^39^ While HFRS has shown validation in acute illness across various patient subsets, establishing associations with factors such as level of independence, functional impairment, QoL, mortality, length of stay, and hospital admission, it is important to note that several other essential elements, such as polypharmacy, mobility, sarcopenia, and hand grip strength, are not accounted for in the score.^13^^,^^40^ A notable gap exists in standardized frailty assessment during acute cardiovascular illness, which may not accurately represent a patient's true frailty level, particularly in cases of critical illnesses like CS, and the timing for evaluating and diagnosing frailty remains elusive. We were only able to assess in-hospital mortality and do not have data on longer-term outcomes that may be relevant, particularly for younger frail individuals. Finally, shared decision-making between clinicians and patients was not captured, potentially leading to selection bias as to whether patients who underwent conservative management did so due to their frailty, multimorbidity, ineligibility, or patient preferences.

## Conclusions

In this nationally representative sample of hospitalizations for AMI-CS, frailty was common and had increased from 2016 to 2020. The presence of frailty was associated with less revascularization and MCS use. Interventions that directly target frailty should be studied to explore their benefit during AMI-CS hospitalizations.

PERSPECTIVES **COMPETENCY IN PATIENT CARE:** AMI-CS is associated with high morbidity and mortality. Frailty is a common comorbidity present during hospitalization for AMI-CS and is associated with less revascularization and MCS use.

**TRANSLATIONAL OUTLOOK IMPLICATIONS:** When presented with AMI-CS, frailty is associated with worse outcomes. Future research targeting frailty during hospitalization for AMI-CS is needed.

## Funding support and author disclosures

Dr Ahmad is a consultant for Cardiovascular Systems Inc and Shockwave and serves on the Medical Advisory Board of Boston Scientific. Dr Bosworth has received research funding through his institution from BeBetter Therapeutics, Boehringer Ingelheim, Esperion, Improved Patient Outcomes, Merck, NHLBI, Novo Nordisk, Otsuka, Sanofi, Veterans Aministration, Elton John Foundation, Hilton foundation, Pfizer; and provides consulting services for Abbott, Esperion, Imatar, Novartis, Sanofi, Vidya, Walmart, and Webmed. He was also on the board of directors of Preventric Diagnostics. Dr Coles has received research support from Merck and consulting with Regenxbio. Dr Damluji has received research funding from the Pepper Scholars Program of the Johns Hopkins University Claude D. Pepper Older Americans Independence Center funded by the National Institute on Aging P30-AG021334 and receives mentored patient-oriented research career development award from the National Heart, Lung, and Blood Institute K23-HL153771-01. Dr Nanna has received research support from the American College of Cardiology Foundation supported by the George F. and Ann Harris Bellows Foundation, the Patient-Centered Outcomes Research Institute (PCORI), the Yale Claude D. Pepper Older Americans Independence Center (P30AG021342), and the National Institute on Aging/National Institutes of Health from R03AG074067 (GEMSSTAR award). All other authors have reported that they have no relationships relevant to the contents of this paper to disclose.
