# Supplementary material for: Association Between Frailty and Management and Outcomes of Acute Myocardial Infarction Complicated by Cardiogenic Shock
Source: JACC Adv. 2024 May 17;3(6):100949. doi: 10.1016/j.jacadv.2024.100949 (PMC11198471; doi:10.1016/j.jacadv.2024.100949)
Supplement: Supplemental Tables 1-6 [file mmc1.docx]

Supplemental Table 1. ICD-10-CM codes used to calculate the Hospital Frailty Risk Score

| **ICD-10-CM** | **Description** | **Awarded Points** |
| --- | --- | --- |
| F00 | Dementia in Alzheimer’s disease | 7.1 |
| G81 | Hemiplegia | 4.4 |
| G30 | Alzheimer’s disease | 4.0 |
| I69 | Sequelae of cerebrovascular disease | 3.7 |
| R29 | Other symptoms and signs involving the nervous and musculoskeletal systems | 3.6 |
| N39 | Other disorders of urinary system | 3.2 |
| F05 | Delirium, not induced by alcohol and other psychoactive substances | 3.2 |
| W19 | Unspecified fall | 3.2 |
| S00 | Superficial injury of head | 3.2 |
| R31 | Unspecified hematuria | 3.0 |
| B96 | Other bacterial agents as the cause of diseases classified to other chapters | 2.9 |
| R41 | Other symptoms and signs involving cognitive functions and awareness | 2.7 |
| R26 | Abnormalities of gait and mobility | 2.6 |
| I67 | Other cerebrovascular diseases | 2.6 |
| R56 | Convulsions, not elsewhere classified | 2.6 |
| R40 | Somnolence, stupor, and coma | 2.5 |
| T83 | Complications of genitourinary prosthetic devices, implants, and grafts | 2.4 |
| S06 | Intracranial injury | 2.4 |
| S42 | Fracture of shoulder and upper arm | 2.3 |
| E87 | Other disorders of fluid, electrolyte, and acid-base balance | 2.3 |
| M25 | Other joint disorders, not elsewhere classified | 2.3 |
| E86 | Volume depletion | 2.3 |
| R54 | Senility | 2.2 |
| Z50 | Care involving use of rehabilitation procedures | 2.1 |
| F03 | Unspecified dementia | 2.1 |
| W18 | Other fall on same level | 2.1 |
| Z75 | Problems related to medical facilities and other healthcare | 2.0 |
| F01 | Vascular dementia | 2.0 |
| S80 | Superficial injury of lower leg | 2.0 |
| L03 | Cellulitis | 2.0 |
| H54 | Blindness and low vision | 1.9 |
| E53 | Deficiency of other B group vitamins | 1.9 |
| Z60 | Problems related to social environment | 1.8 |
| G20 | Parkinson’s disease | 1.8 |
| R55 | Syncope and collapse | 1.8 |
| S22 | Fracture of rib(s), sternum, and thoracic spine | 1.8 |
| K59 | Other functional intestinal disorders | 1.8 |
| N17 | Acute renal failure | 1.8 |
| L89 | Decubitus ulcer | 1.7 |
| Z22 | Carrier of infectious disease | 1.7 |
| B95 | Streptococcus and staphylococcus as the causes of diseases classified to other chapters | 1.7 |
| L97 | Ulcer of lower limb, not elsewhere classified | 1.6 |
| R44 | Other symptoms and signs involving general sensations and perceptions | 1.6 |
| K26 | Duodenal ulcer | 1.6 |
| I95 | Hypotension | 1.6 |
| N19 | Unspecified renal failure | 1.6 |
| A41 | Other septicemia | 1.6 |
| Z87 | Personal history of other diseases and conditions | 1.5 |
| J96 | Respiratory failure, not elsewhere classified | 1.5 |
| X59 | Exposure to unspecified factor | 1.5 |
| M19 | Other arthrosis | 1.5 |
| G40 | Epilepsy | 1.5 |
| M81 | Osteoporosis without pathological fracture | 1.4 |
| S72 | Fracture of femur | 1.4 |
| S32 | Fracture of lumbar spine and pelvis | 1.4 |
| E16 | Other disorders of pancreatic internal secretion | 1.4 |
| R94 | Abnormal results of function studies | 1.4 |
| N18 | Chronic renal failure | 1.4 |
| R33 | Retention of urine | 1.3 |
| R69 | Unknown and unspecified causes of morbidity | 1.3 |
| N28 | Other disorders of kidney and ureter, not elsewhere classified | 1.3 |
| R32 | Unspecified urinary incontinence | 1.2 |
| G31 | Other degenerative diseases of nervous system, not elsewhere classified | 1.2 |
| Y95 | Nosocomial condition | 1.2 |
| S09 | Other and unspecified injuries of head | 1.2 |
| R45 | Symptoms and signs involving emotional state | 1.2 |
| G45 | Transient cerebral ischemic attacks and related symptoms | 1.2 |
| Z74 | Problems related to care-provider dependency | 1.1 |
| M79 | Other soft tissue disorders, not elsewhere classified | 1.1 |
| W06 | Fall involving bed | 1.1 |
| S01 | Open wound of head | 1.1 |
| A04 | Other bacterial intestinal infections | 1.1 |
| A09 | Diarrhea and gastroenteritis of presume infectious origin | 1.1 |
| J18 | Pneumonia, organism unspecified | 1.1 |
| J69 | Pneumonitis due to solids and liquids | 1.0 |
| R47 | Speech disturbances, not elsewhere classified | 1.0 |
| E55 | Vitamin D deficiency | 1.0 |
| Z93 | Artificial opening status | 1.0 |
| R02 | Gangrene, not elsewhere classified | 1.0 |
| R63 | Symptoms and signs concerning food and fluid intake | 0.9 |
| H91 | Other hearing loss | 0.9 |
| W10 | Fall on and from stairs and steps | 0.9 |
| W01 | Fall on same level from slipping, tripping, and stumbling | 0.9 |
| E05 | Thyrotoxicosis | 0.9 |
| M41 | Scoliosis | 0.9 |
| R13 | Dysphagia | 0.8 |
| Z99 | Dependence on enabling machines and deices | 0.8 |
| U80 | Agent resistant to penicillin and related antibiotics | 0.8 |
| M80 | Osteoporosis with pathological fracture | 0.8 |
| K92 | Other diseases of digestive system | 0.8 |
| I63 | Cerebral infarction | 0.8 |
| N20 | Calculus of kidney and ureter | 0.7 |
| F10 | Mental and behavioral disorders due to use of alcohol | 0.7 |
| Y84 | Other medical procedures as the cause of abnormal reaction of the patient | 0.7 |
| R00 | Abnormalities of heartbeat | 0.7 |
| J22 | Unspecified acute lower respiratory infection | 0.7 |
| Z73 | Problems related to life-management difficulty | 0.6 |
| R79 | Other abnormal findings of blood chemistry | 0.6 |
| Z91 | Personal history of risk-factors, not elsewhere classified | 0.5 |
| S51 | Open wound of forearm | 0.5 |
| F32 | Depressive episode | 0.5 |
| M48 | Spinal stenosis | 0.5 |
| E83 | Disorders of mineral metabolism | 0.4 |
| M15 | Polyarthrosis | 0.4 |
| D64 | Other anemias | 0.4 |
| L08 | Other local infections of skin and subcutaneous tissue | 0.4 |
| R11 | Nausea and vomiting | 0.3 |
| K52 | Other noninfective gastroenteritis and colitis | 0.3 |
| R50 | Fever of unknown origin | 0.1 |

Supplemental Table 2. List of the ICD-10-CM codes used

| **Diagnosis** | **ICD-10 Code** |
| --- | --- |
| ST-elevation myocardial infarction | I21.0, I21.1, I21.2, I21.3, I21.9, I22.0, I22.1, I22.8, I22.9 |
| Non-ST-elevation myocardial infarction | I21.4, I22.2 |
| Cardiogenic shock | R57.0 |
| Smoking | F17, T65, Z72.0, O99.33, Z87.891 |
| Hypertension | I10 |
| Diabetes mellitus | E08, E10, E11, E13 |
| Hyperlipidemia | E78 |
| Obesity | E66 |
| Heart failure | I09.81, I11.0, I13.0, I31.2, I50 |
| Chronic ischemic heart disease | I25 |
| Atrial fibrillation | I48.0, I48.1, I48.2, I48.91 |
| Valvular heart disease | I34, I35, I36, I37 |
| Peripheral artery disease | I70 |
| Previous PCI | Z98.61 |
| Previous CABG | Z95.1 |
| Previous stroke | I69, Z86.73 |
| Previous pacemaker | Z95.0 |
| Chronic obstructive pulmonary disease | J41, J42, J43, J44 |
| Pulmonary hypertension | I27.0, I27.2 |
| Chronic kidney disease | N18 |
| End-stage renal disease | N18.6 |
| Liver cirrhosis | K70.2, K70.3, K71.7, K74, K76.1, P78.81, E83.110 |
| History of malignancy | Z85 |
| Deficiency anemia | D50, D51, D52, D53 |
| Malnutrition | E43, E44, E46 |
| Major depression | F33 |
| Palliative care consult | Z51.5, Z71.89 |
| Do not resuscitate | Z66 |
| Coronary revascularization* | 027034, 027035, 027036, 027037, 027044, 027045, 027046, 027047, 027134, 027135, 027136, 027137, 027144, 027145, 027146, 027147, 027234, 027235, 027236, 027237, 027244, 027245, 027246, 027247,  027334, 027335, 027336, 027337, 027344, 027345, 027346, 027347, 02703D, 02703E, 02703F, 02703G, 02704D, 02704E, 02704F, 02704G,  02713D, 02713E, 02713F, 02713G, 02714D, 02714E, 02714F, 02714G,  02723D, 02723E, 02723F, 02723G, 02724D, 02724E, 02724F, 02724G,  02733D, 02733E, 02733F, 02733G, 02734D, 02734E, 02734F, 02734G, 02703Z, 02704Z, 02713Z, 02714Z, 02723Z, 02724Z, 02733Z, 02734Z, 02100, 02110, 02120, 02130 |
| Mechanical circulatory support* | 5A02110, 5A02210, 5A02116, 5A0211D, 5A02216, 5A0221D, 02HA3, 02HA4, 02HA0QZ, 5A15 |
| Intracranial hemorrhage | I60, I61, I62, S06.3, S06.4, S06.5, S06.6 |
| Gastrointestinal hemorrhage | I85.01, I85.11, K25.0, K25.2, K25.4, K25.6, K26.0, K26.2, K26.4, K26.6, K27.0, K27.2, K27.4, K27.6, K28.0, K28.2, K28.4, K28.6, K29.01, K29.21, K29.31, K29.41, K29.51, K29.61, K29.71, K29.81, K29.91, K62.5, K92.0, K92.1, K92.2 |
| Acute kidney injury | N17 |
| Delirium | F05, R41.0, R41.82 |

*ICD-10-PCS codes

Abbreviations: CABG, coronary artery bypass graft; ECMO, extracorporeal membranous oxygenation; LVAD, left ventricular assist device; ICD-10-CM, International Classification of Diseases, Tenth Revision, Clinical Modification; ICD-10-PCS, International Classification of Diseases, Tenth Revision, Procedure Coding System; PCI, percutaneous coronary intervention

Supplemental Table 3. Sensitivity analysis of revascularization or mechanical circulatory support

| **Outcome** | **Frailty (+)** | **Frailty (-)** | **Crude Odds Ratio** | ***P*-value** | **Adjusted Odds Ratio**^a^ | ***P*-value** |
| --- | --- | --- | --- | --- | --- | --- |
| In-hospital mortality (%) | 33.5 | 20.5 | 1.95 (1.81-2.11) | <.001 | 2.13 (1.96-2.31) | <.001 |
| Do-not resuscitate (%) | 20.0 | 8.2 | 2.80 (2.2-3.12) | <.001 | 2.65 (2.36-2.96) | <.001 |
| Palliative care consult (%) | 14.5 | 5.1 | 3.15 (2.77-3.59) | <.001 | 2.89 (2.52-3.32) | <.001 |
| Skilled nursing facility (%) | 29.1 | 11.6 | 3.15 (2.88-3.44) | <.001 | 2.34 (2.13-2.57) | <.001 |
| Intracranial hemorrhage (%) | 1.7 | 0.3 | 6.09 (3.63-10.19) | <.001 | 5.96 (3.48-10.21) | <.001 |
| Gastrointestinal hemorrhage (%) | 9.1 | 2.0 | 4.99 (4.11-6.06) | <.001 | 4.53 (3.70-5.55) | <.001 |
| Acute kidney injury (%) | 70.5 | 17.6 | 11.16 (10.31-12.08) | <.001 | 11.60 (10.67-12.62) | <.001 |
| Delirium (%) | 7.0 | 0.5 | 15.89 (10.81-23.36) | <.001 | 13.34 (9.03-19.72) | <.001 |
| Length of stay (days ± SD) | 13.1 ± 13.2 | 3.5 ± 3.6 | 6.65 (6.28-7.01)^b^ | <.001 | 4.62 (4.27-4.96)^c^ | <.001 |
| Total hospital cost ($ ± SD) | 87,764 ± 76,750 | 52,767 ± 43,093 | 34,995 (32,851-37,140)^b^ | <.001 | 26,082 (23,967-28,198)^c^ | <.001 |

^a^Adjusted for age, sex, race, smoking, diabetes mellitus, hyperlipidemia, obesity, heart failure, chronic ischemic heart disease, atrial fibrillation, peripheral artery disease, previous CABG, previous stroke, pulmonary hypertension, end-stage renal disease, liver cirrhosis, deficiency anemia, malnutrition, dementia, major depression, and ST-elevation myocardial infarction

^b^Crude mean difference with 95% confidence interval

^c^Adjusted mean difference with 95% confidence interval

Abbreviations: AMI, acute myocardial infarction; CABG, coronary artery bypass graft; CS, cardiogenic shock; MCS, mechanical circulatory support; SD, standard deviation

Supplemental Table 4. Subgroup analysis according to younger (age <65 years) and older (≥65 years) patients

| **Age Group** | **Outcome** | **Frailty (+)** | **Frailty (-)** | **Adjusted Odds Ratio**^a^ | ***P*-value** |
| --- | --- | --- | --- | --- | --- |
| **Age <65 years**  (N=104,025) | Sample size | 66,680 | 37,345 | - | - |
|  | In-hospital mortality (%) | 33.5 | 15.4 | 2.93 (2.70-3.17) | <.001 |
|  | Do-not resuscitate (%) | 18.4 | 5.7 | 3.60 (3.21-4.05) | <.001 |
|  | Palliative care consult (%) | 14.8 | 4.2 | 3.69 (3.24-4.22) | <.001 |
|  | Skilled nursing facility (%) | 19.9 | 5.6 | 3.13 (2.80-3.51) | <.001 |
|  | Revascularization (%) | 52.1 | 75.2 | 0.47 (0.44-0.51) | <.001 |
|  | MCS (%) | 40.9 | 46.3 | 0.90 (0.85-0.97) | 0.002 |
|  | Intracranial hemorrhage (%) | 2.4 | 0.3 | 7.73 (4.91-12.17) | <.001 |
|  | Gastrointestinal hemorrhage (%) | 8.8 | 2.0 | 4.25 (3.54-5.09) | <.001 |
|  | Acute kidney injury (%) | 69.5 | 16.9 | 12.44 (11.54-13.42) | <.001 |
|  | Delirium (%) | 4.8 | 0.2 | 18.44 (11.50-29.59) | <.001 |
|  | Length of stay (days ± SD) | 12.8 ± 14.5 | 6.0 ± 6.5 | 4.50 (4.16-4.85)^b^ | <.001 |
|  | Total hospital cost ($ ± SD) | 70,968 ± 80,174 | 41,171 ± 42,880 | 21,783 (19,798-23,767)^b^ | <.001 |
| **Age ≥65 years**  (N=179,745) | Sample size | 134,290 | 45,455 | - | - |
|  | In-hospital mortality (%) | 43.3 | 31.9 | 1.82 (1.73-1.92) | <.001 |
|  | Do-not resuscitate (%) | 35.3 | 20.0 | 2.04 (1.92-2.17) | <.001 |
|  | Palliative care consult (%) | 24.2 | 12.5 | 2.02 (1.88-2.17) | <.001 |
|  | Skilled nursing facility (%) | 29.2 | 14.9 | 1.90 (1.78-2.03) | <.001 |
|  | Revascularization (%) | 45.1 | 63.6 | 0.961 (0.958-0.964) | <.001 |
|  | MCS (%) | 30.6 | 38.7 | 0.88 (0.83-0.93) | <.001 |
|  | Intracranial hemorrhage (%) | 1.4 | 0.4 | 4.36 (3.03-6.29) | <.001 |
|  | Gastrointestinal hemorrhage (%) | 8.4 | 2.6 | 3.28 (2.85-3.79) | <.001 |
|  | Acute kidney injury (%) | 72.3 | 22.2 | 10.19 (9.60-10.82) | <.001 |
|  | Delirium (%) | 6.0 | 0.5 | 11.05 (8.28-14.75) | <.001 |
|  | Length of stay (days ± SD) | 10.1 ± 10.9 | 5.5 ± 5.8 | 3.42 (3.19-3.65)^b^ | <.001 |
|  | Total hospital cost ($ ± SD) | 51,674 ± 56,869 | 35,923 ± 33,115 | 14,261 (13,036-15,485)^b^ | <.001 |

^a^Adjusted for age, sex, race, smoking, diabetes mellitus, hyperlipidemia, obesity, heart failure, chronic ischemic heart disease, atrial fibrillation, peripheral artery disease, previous CABG, previous stroke, pulmonary hypertension, end-stage renal disease, liver cirrhosis, deficiency anemia, malnutrition, dementia, major depression, and ST-elevation myocardial infarction

^b^Adjusted mean difference with 95% confidence interval

Abbreviations: CABG, coronary artery bypass graft; MCS, mechanical circulatory support; SD, standard deviation

Supplemental Table 5. Subgroup analysis stratified to STEMI and NSTEMI

| **Presentation** | **Outcome** | **Frailty (+)** | **Frailty (-)** | **Adjusted Odds Ratio**^a^ | ***P*-value** |
| --- | --- | --- | --- | --- | --- |
| **STEMI**  (N=138,350) | Sample size | 86,886 | 51,465 | - | - |
|  | In-hospital mortality (%) | 42.6 | 25.2 | 2.31 (2.18-2.45) | <.001 |
|  | Do-not resuscitate (%) | 29.3 | 11.9 | 2.76 (2.56-2.97) | <.001 |
|  | Palliative care consult (%) | 20.7 | 7.5 | 2.78 (2.55-3.03) | <.001 |
|  | Skilled nursing facility (%) | 22.7 | 7.8 | 2.65 (2.44-2.88) | <.001 |
|  | Revascularization (%) | 63.7 | 79.2 | 0.55 (0.52-0.58) | <.001 |
|  | MCS (%) | 46.2 | 47.1 | 1.02 (0.97-1.08) | 0.431 |
|  | Intracranial hemorrhage (%) | 2.0 | 0.3 | 7.30 (4.93-10.82) | <.001 |
|  | Gastrointestinal hemorrhage (%) | 9.1 | 2.2 | 3.94 (3.40-4.57) | <.001 |
|  | Acute kidney injury (%) | 69.6 | 16.1 | 12.30 (11.51-13.15) | <.001 |
|  | Delirium (%) | 5.6 | 0.5 | 11.30 (8.42-15.15) | <.001 |
|  | Length of stay (days ± SD) | 10.1 ± 12.0 | 6.0 ± 6.5 | 4.04 (3.81-4.28)^b^ | <.001 |
|  | Total hospital cost ($ ± SD) | 61,206 ± 69,464 | 36,389 ± 35,334 | 20,326 (18,898-21,755)^b^ | <.001 |
| **NSTEMI**  (N=145,420) | Sample size | 114,085 | 31,335 | - | - |
|  | In-hospital mortality (%) | 38.1 | 23.3 | 2.00 (1.87-2.13) | <.001 |
|  | Do-not resuscitate (%) | 30.0 | 16.3 | 1.93 (1.78-2.08) | <.001 |
|  | Palliative care consult (%) | 21.4 | 10.8 | 1.98 (1.80-2.16) | <.001 |
|  | Skilled nursing facility (%) | 28.7 | 15.6 | 1.85 (1.71-2.00) | <.001 |
|  | Revascularization (%) | 35.1 | 51.8 | 0.988 (0.986-0.990) | <.001 |
|  | MCS (%) | 24.8 | 34.0 | 0.72 (0.67-0.77) | <.001 |
|  | Intracranial hemorrhage (%) | 1.5 | 0.4 | 3.85 (2.58-5.76) | <.001 |
|  | Gastrointestinal hemorrhage (%) | 8.1 | 2.5 | 3.16 (2.66-3.76) | <.001 |
|  | Acute kidney injury (%) | 72.7 | 25.7 | 9.66 (9.01-10.35) | <.001 |
|  | Delirium (%) | 5.6 | 0.3 | 18.12 (11.37-28.86) | <.001 |
|  | Length of stay (days ± SD) | 11.6 ± 12.5 | 7.4 ± 7.0 | 3.59 (3.27-3.90)^b^ | <.001 |
|  | Total hospital cost ($ ± SD) | 55,691 ± 63,413 | 41,442 ± 41,640 | 13,441 (11,808-15,073)^b^ | <.001 |

^a^Adjusted for age, sex, race, smoking, diabetes mellitus, hyperlipidemia, obesity, heart failure, chronic ischemic heart disease, atrial fibrillation, peripheral artery disease, previous CABG, previous stroke, pulmonary hypertension, end-stage renal disease, liver cirrhosis, deficiency anemia, malnutrition, dementia, and major depression

^b^Adjusted mean difference with 95% confidence interval

Abbreviations: CABG, coronary artery bypass graft; MCS, mechanical circulatory support; NSTEMI, non-ST-elevation myocardial infarction; SD, standard deviation; STEMI, ST-elevation myocardial infarction

Supplemental Table 6. Impact of revascularization or mechanical circulatory support on in-hospital outcomes

|  | **Outcome** | **Revascularization (+)** | **Revascularization (-)** | **Adjusted Odds Ratio**^a^ | ***P*-value** |
| --- | --- | --- | --- | --- | --- |
| **Frail** | Sample size | 95,340 | 105,630 | - | - |
|  | In-hospital mortality (%) | 29.5 | 49.5 | 0.44 (0.42-0.46) | <.001 |
|  | Do-not resuscitate (%) | 19.2 | 39.2 | 0.41 (0.39-0.43) | <.001 |
|  | Palliative care consult (%) | 13.4 | 28.0 | 0.44 (0.42-0.47) | <.001 |
|  | Skilled nursing facility (%) | 29.7 | 22.8 | 1.64 (1.56-1.72) | <.001 |
|  | Intracranial hemorrhage (%) | 1.4 | 2.0 | 0.73 (0.63-0.86) | <.001 |
|  | Gastrointestinal hemorrhage (%) | 8.2 | 8.9 | 0.95 (0.88-1.03) | 0.191 |
|  | Acute kidney injury (%) | 68.5 | 74.0 | 0.73 (0.70-0.77) | <.001 |
|  | Delirium (%) | 6.6 | 4.7 | 1.57 (0.43-1.72) | <.001 |
|  | Length of stay (days ± SD) | 12.6 ± 12.2 | 9.5 ± 12.2 | 3.39 (3.16-3.62)^b^ | <.001 |
|  | Total hospital cost ($ ± SD) | 74,998 ± 67,541 | 42,800 ± 60,967 | 32,558 (31,336-33,779)^b^ | <.001 |
|  | **Outcome** | **MCS (+)** | **MCS (-)** | **Adjusted Odds Ratio**^a^ | ***P*-value** |
|  | Sample size | 68,395 | 132,575 |  |  |
|  | In-hospital mortality (%) | 36.6 | 41.8 | 0.91 (0.87-0.96) | <.001 |
|  | Do-not resuscitate (%) | 21.9 | 33.8 | 0.68 (0.64-0.71) | <.001 |
|  | Palliative care consult (%) | 16.6 | 23.4 | 0.76 (0.72-0.81) | <.001 |
|  | Skilled nursing facility (%) | 27.0 | 25.6 | 1.19 (1.13-1.25) | <.001 |
|  | Intracranial hemorrhage (%) | 1.8 | 1.7 | 1.04 (0.88-1.22) | 0.670 |
|  | Gastrointestinal hemorrhage (%) | 9.1 | 8.2 | 1.12 (1.04-1.21) | 0.003 |
|  | Acute kidney injury (%) | 72.4 | 70.8 | 1.01 (0.96-1.06) | 0.806 |
|  | Delirium (%) | 6.7 | 5.0 | 1.42 (1.29-1.56) | <.001 |
|  | Length of stay (days ± SD) | 13.2 ± 14.2 | 9.8 ± 11.0 | 2.92 (2.68-3.16)^b^ | <.001 |
|  | Total hospital cost ($ ± SD) | 88,599 ± 86,071 | 42,328 ± 45,645 | 42,741 (41,491-43,990)^b^ | <.001 |
|  | **Outcome** | **Revascularization or MCS (+)** | **Revascularization or MCS (-)** | **Adjusted Odds Ratio**^a^ | ***P*-value** |
|  | Sample size | 111,140 | 89,830 |  |  |
|  | In-hospital mortality (%) | 32.0 | 50.0 | 0.51 (0.48-0.53) | <.001 |
|  | Do-not resuscitate (%) | 20.5 | 41.1 | 0.43 (0.41-0.45) | <.001 |
|  | Palliative care consult (%) | 14.9 | 28.8 | 0.50 (0.47-0.52) | <.001 |
|  | Skilled nursing facility (%) | 28.3 | 23.3 | 1.51 (1.44-1.59) | <.001 |
|  | Intracranial hemorrhage (%) | 1.5 | 1.9 | 0.79 (0.68-0.92) | 0.003 |
|  | Gastrointestinal hemorrhage (%) | 8.3 | 8.8 | 0.97 (0.90-1.05) | 0.452 |
|  | Acute kidney injury (%) | 70.0 | 73.1 | 0.81 (0.77-0.85) | <.001 |
|  | Delirium (%) | 6.5 | 4.5 | 1.63 (1.48-1.80) | <.001 |
|  | Length of stay (days ± SD) | 12.7 ± 13.0 | 8.8 ± 11.0 | 3.97 (3.74-4.20) | <.001 |
|  | Total hospital cost ($ ± SD) | 77,328 ± 75,604 | 34,256 ± 41,144 | 42,419 (41,213-43,625) | <.001 |
|  | **Outcome** | **Revascularization (+)** | **Revascularization (-)** | **Adjusted Odds Ratio**^a^ | ***P*-value** |
| **Non-frail** | Sample size | 56,990 | 25,810 |  | - |
|  | In-hospital mortality (%) | 16.6 | 41.7 | 0.33 (0.30-0.35) | <.001 |
|  | Do-not resuscitate (%) | 7.3 | 27.3 | 0.29 (0.26-0.33) | <.001 |
|  | Palliative care consult (%) | 4.3 | 18.6 | 0.28 (0.25-0.32) | <.001 |
|  | Skilled nursing facility (%) | 10.8 | 10.6 | 1.54 (1.37-1.74) | <.001 |
|  | Intracranial hemorrhage (%) | 0.2 | 0.5 | 0.50 (0.28-0.91) | 0.023 |
|  | Gastrointestinal hemorrhage (%) | 2.1 | 2.9 | 0.85 (0.68-1.07) | 0.162 |
|  | Acute kidney injury (%) | 16.3 | 27.5 | 0.60 (0.55-0.65) | <.001 |
|  | Delirium (%) | 0.4 | 0.3 | 1.45 (0.80-2.63) | 0.221 |
|  | Length of stay (days ± SD) | 6.1 ± 5.9 | 4.9 ± 6.6 | 1.70 (1.49-1.88)^b^ | <.001 |
|  | Total hospital cost ($ ± SD) | 44,150 ± 36,358 | 25,350 ± 38,098 | 20,764 (19,540-21,988)^b^ | <.001 |
|  | **Outcome** | **MCS (+)** | **MCS (-)** | **Adjusted Odds Ratio**^a^ | ***P*-value** |
|  | Sample size | 34,895 | 47,905 |  |  |
|  | In-hospital mortality (%) | 23.1 | 25.5 | 1.07 (0.99-1.16) | 0.112 |
|  | Do-not resuscitate (%) | 9.5 | 16.5 | 0.69 (0.62-0.77) | <.001 |
|  | Palliative care consult (%) | 6.4 | 10.5 | 0.75 (0.66-0.85) | <.001 |
|  | Skilled nursing facility (%) | 11.0 | 10.5 | 1.29 (1.16-1.43) | <.001 |
|  | Intracranial hemorrhage (%) | 0.4 | 0.3 | 1.46 (0.83-2.57) | 0.189 |
|  | Gastrointestinal hemorrhage (%) | 2.0 | 2.6 | 0.81 (0.66-1.01) | 0.061 |
|  | Acute kidney injury (%) | 18.9 | 20.4 | 0.89 (0.82-0.96) | 0.004 |
|  | Delirium (%) | 0.4 | 0.4 | 1.10 90.68-1.78) | 0.709 |
|  | Length of stay (days ± SD) | 6.3 ± 6.8 | 5.3 ± 5.6 | 0.94 (0.76-1.12)^b^ | <.001 |
|  | Total hospital cost ($ ± SD) | 51,198 ± 46,807 | 28,887 ± 26,077 | 21,188 (20,089-22,287)^b^ | <.001 |
|  | **Outcome** | **Revascularization or MCS (+)** | **Revascularization or MCS (-)** | **Adjusted Odds Ratio**^a^ | ***P*-value** |
|  | Sample size | 63,025 | 19,775 |  |  |
|  | In-hospital mortality (%) | 18.4 | 43.7 | 0.36 (0.33-0.40) | <.001 |
|  | Do-not resuscitate (%) | 8.2 | 30.8 | 0.30 (0.27-0.33) | <.001 |
|  | Palliative care consult (%) | 5.1 | 20.5 | 0.32 (0.28-0.36) | <.001 |
|  | Skilled nursing facility (%) | 10.6 | 11.3 | 1.50 (1.32-1.71) | <.001 |
|  | Intracranial hemorrhage (%) | 0.3 | 0.4 | 0.91 (0.47-1.76) | 0.776 |
|  | Gastrointestinal hemorrhage (%) | 2.1 | 3.2 | 0.79 (0.62-1.01) | 0.061 |
|  | Acute kidney injury (%) | 17.1 | 28.3 | 0.61 (0.56-0.67) | <.001 |
|  | Delirium (%) | 0.4 | 0.4 | 1.20 (0.65-2.24) | 0.558 |
|  | Length of stay (days ± SD) | 6.0 ± 6.2 | 4.7 ± 5.9 | 1.79 (1.58-2.00)^b^ | <.001 |
|  | Total hospital cost ($ ± SD) | 44,106 ± 39,388 | 19,752 ± 25,007 | 26,299 (24,985-27,614)^b^ | <.001 |

^a^Adjusted for age, sex, race, smoking, diabetes mellitus, hyperlipidemia, obesity, heart failure, chronic ischemic heart disease, atrial fibrillation, peripheral artery disease, previous CABG, previous stroke, pulmonary hypertension, end-stage renal disease, liver cirrhosis, deficiency anemia, malnutrition, dementia, and major depression

^b^Adjusted mean difference with 95% confidence interval

Abbreviations: CABG, coronary artery bypass graft; MCS, mechanical circulatory support; NSTEMI, non-ST-elevation myocardial infarction; SD, standard deviation; STEMI, ST-elevation myocardial infarction
